# Peer Mentoring Program for Informal Caregivers of Homebound Individuals With Advanced Parkinson Disease (Share the Care): Protocol for a Single-Center, Crossover Pilot Study

**DOI:** 10.2196/34750

**Published:** 2022-05-26

**Authors:** Jori E Fleisher, Faizan Akram, Jeanette Lee, Ellen C Klostermann, Serena P Hess, Erica Myrick, Melissa Levin, Bichun Ouyang, Jayne Wilkinson, Deborah A Hall, Joshua Chodosh

**Affiliations:** 1 Rush University Medical Center Chicago, IL United States; 2 Western Michigan University Homer Stryker M D School of Medicine Kalamazoo, MI United States; 3 Chicago Medical School, Rosalind Franklin University North Chicago, IL United States; 4 Department of Neurology University of Pennsylvania Pennsylvania, PA United States; 5 Division of Geriatric Medicine and Palliative Care Department of Medicine New York University School of Medicine New York, NY United States; 6 Medicine Service Veterans Affairs New York Harbor Healthcare System New York, NY United States

**Keywords:** Parkinson disease, carer, caregiving, care partner, peer mentor, peer support, depression, anxiety, caregiver strain, volunteer, intervention

## Abstract

**Background:**

Homebound individuals with advanced Parkinson disease (PD) require intensive caregiving, the majority of which is provided by informal, family caregivers. PD caregiver strain is an independent risk factor for institutionalization. There are currently no effective interventions to support advanced PD caregivers. Studies in other neurologic disorders, however, have demonstrated the potential for peer mentoring interventions to improve caregiver outcomes. In the context of an ongoing trial of interdisciplinary home visits, we designed and piloted a nested trial of caregiver peer mentoring for informal caregivers of individuals with advanced PD.

**Objective:**

The aim of this study was to test the feasibility of peer mentoring for caregivers of homebound individuals with advanced PD and to evaluate its effects on anxiety, depression, and caregiver strain.

**Methods:**

This was a single-center, 16-week pilot study of caregiver peer mentoring nested within a year-long controlled trial of interdisciplinary home visits. We recruited 34 experienced former or current family caregivers who completed structured mentor training. Caregivers enrolled in the larger interdisciplinary home visit trial consented to receive 16 weeks of weekly, one-to-one peer mentoring calls with a trained peer mentor. Weekly calls were guided by a curriculum on advanced PD management and caregiver support. Fidelity to and satisfaction with the intervention were gathered via biweekly study diaries. Anxiety, depression, and caregiver strain were measured pre- and postmentoring intervention at home visits 2 and 3.

**Results:**

Enrollment and peer-mentor training began in 2018, and 65 caregivers enrolled in the overarching trial. The majority of mentors and mentees were White, female spouses or partners of individuals with PD; mentors had a mean of 8.7 (SD 6.4) years of caregiving experience, and 33 mentors were matched with at least 1 mentee.

**Conclusions:**

This is the first study of caregiver peer mentoring in PD and may establish an adaptable and sustainable model for disease-specific caregiver interventions in PD and other neurodegenerative diseases.

**Trial Registration:**

ClinicalTrials.gov NCT03189459; http://clinicaltrials.gov/ct2/show/NCT03189459

**International Registered Report Identifier (IRRID):**

DERR1-10.2196/34750

## Introduction

### Background

Parkinson disease (PD) affects over 1 million individuals in the United States, with a projected 77% increase in prevalence by 2030 [1,2]. Although PD is classified as a movement disorder, the nonmotor and neuropsychiatric symptoms and complications frequently overshadow mobility concerns as the disease progresses [3-6]. Indeed, the leading causes of hospitalization in PD are falls, urinary incontinence or infection, dehydration, and neuropsychiatric changes—such as dementia, hallucinations, delusions, agitation, and depression. Many of these hospitalization triggers are preventable or treatable at home if recognized and addressed promptly, which requires a knowledgeable and observant caregiver [7]. Once hospitalized or institutionalized, people with PD suffer excessive iatrogenic morbidity and mortality.

A critical, understudied, and independent risk factor in both hospitalization and institutionalization in PD is caregiver strain [8-10]. Ample evidence links PD caregiver strain to acute health care utilization for the patient and to adverse caregiver health consequences [3,11-13]. Furthermore, caregiver strain is higher in PD than in many other neurodegenerative conditions, likely due to the complexity and synergistic effects of the motor, nonmotor, and cognitive symptoms [13-16]. However, few interventions have targeted caregiver strain in this population despite significant work highlighting unmet needs for education, prognostic counseling, and support for PD caregivers [17-19]. The lack of evidence-based caregiver interventions is not limited to PD. In 2020, the National Institute on Aging published a systematic review on behavioral interventions for individuals with dementia and their caregivers, concluding that while an intensive, multicomponent intervention may improve caregiver depression at 6 months, the majority of other interventions and care models demonstrated minimal positive effect [20]. Many caregiver interventions also rely on costly measures performed by the medical team that are both time- and effort-intensive [21].

In prior work, we developed interdisciplinary home visits for homebound individuals and their caregivers affected by advanced PD and related disorders [22]. Home visits appeared to disentangle the expected parallel declines in quality of life and disease severity, such that while mobility worsened over 1 year, quality of life did not follow a similar trajectory [23]. Despite this promising effect seen in patients, among those patients who had a caregiver who participated in longitudinal assessments, caregiver strain worsened over 1 year. We identified key challenges faced by these caregivers from our own cohort and from published studies, including an unmet need for education and social connection among caregivers with similar experiences [24-27].

An intriguing approach to supporting individuals with chronic disease pairs a current caregiver with an experienced past caregiver who is trained as a peer mentor. The mentor offers one-to-one support of the current caregiver (mentee) by providing guidance, resources, and a relationship for problem solving and encouragement [28]. The structure and outcome measures of these studies have varied widely, yet some have improved caregiver strain and confidence [29-31]. Early studies matched Alzheimer disease and Alzheimer disease–related dementias (AD/ADRD) caregivers with peer mentors to bolster coping skills and social support [30-32]. Qualitative data showed benefits for caregivers and mentors although quantitative results were equivocal, likely due to variable implementation [30,33]. Subsequent successful models of peer mentoring have been tested in individuals with end-stage renal disease and advanced cancer, and among older adults to encourage physical activity [34-38]. However, there is a knowledge gap regarding the content, logistics, and implementation of peer mentorship. In anticipation of the proof-of-concept study of peer mentoring among advanced PD caregivers described here, we surveyed a convenience sample of past and current advanced PD caregivers regarding their interest in this kind of program. Over 83% (15/18) expressed interest in being a peer mentor, 50% (9/18) reported prior use and comfort with video chat apps to facilitate mentoring calls, and among those without prior experience, 78% (14/18) indicated willingness to try the technology. We also reviewed notes from 29 PD caregiver support group sessions to identify themes for a PD-specific peer mentoring program.

### Objectives

In response to the success of our home visit pilot program, we designed a larger, controlled trial of interdisciplinary home visits for homebound patient-caregiver dyads with advanced PD versus usual care [39]. In recognition of the dearth of existing interventions, our own data demonstrating progression of strain despite home visits, and interest among caregivers for their own caregiver-directed intervention, we developed a caregiver peer mentoring program entitled Share the Care, nested within the larger year-long home visit trial. Our specific aim for Share the Care was to compare the effects of home visits plus caregiver peer mentoring versus home visits alone on caregiver mood in a single-cohort, crossover design. We hypothesized that caregivers participating in both home visits and nested peer mentoring would have decreased depression and anxiety after 16 weeks of a structured peer mentoring intervention as measured by the change in the Hospital Anxiety and Depression Scale (HADS) [40]. Here, we describe the study design and implementation of Share the Care. We describe the mentor training process and curriculum as well as the structure of peer mentoring and present the baseline characteristics of caregiver peer mentors and mentees, respectively. We also outline the challenges identified in the recruitment and retention of both mentors and mentees that will shape future iterations of this model of caregiver support.

## Methods

### Study Setting, Design, and Recruitment

**Figure 1:**
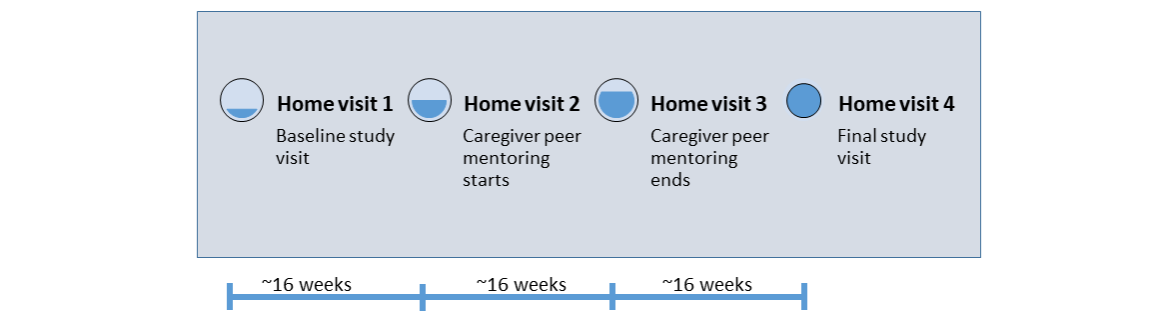
Study design.

This is a pragmatic, single-center, 16-week, single-cohort, crossover study of peer mentoring, nested in a controlled trial of interdisciplinary home visits for individuals with advanced PD and their caregivers (IN-HOME-PD). The full details of IN-HOME-PD have been published previously [39]. Briefly, IN-HOME-PD patient and caregiver dyads received 4 home protocol-driven home visits from a nurse and a social worker, each enhanced by a real-time telehealth connection with a movement disorders specialist over the course of approximately one year. The study described herein occurred between the second and third home visits for each dyad as illustrated in Figure 1.

The recruitment target for IN-HOME-PD was 65 patient-caregiver dyads, with recruitment beginning in the first quarter of 2018 and ending in the fourth quarter of 2019. Patient-caregiver dyads were recruited from the Rush University Medical Center in Chicago, Illinois, and the caregiver member of each dyad served as the mentee in the nested study described here.

Peer mentor recruitment took place between the second quarter of 2018 and the first quarter of 2019. Recruitment was multipronged in order to identify caregivers with both the experience, time, and temperament necessary to serve as a peer mentor. First, we searched the electronic medical record (EMR) and a voluntary registry of current and former patients maintained at Rush’s Parkinson’s Disease and Movement Disorders Program, filtering for patients with a PD diagnosis, seen within 3 years, and with a record of death. These criteria were selected to ensure that the respective caregiver would be familiar with advanced PD, close to having received care at Rush, yet not actively caring for their loved one such that mentoring would be onerous or exacerbate bereavement. All potential mentors identified through the EMR or database were discussed with the previously treating neurologist prior to the study team contacting them. Second, we approached neurologists at Rush’s Parkinson’s Disease and Movement Disorders Program for provider-generated recommendations of caregivers, including those who were still actively caring for their loved ones but might have had the capacity for mentoring. Third, the Rush Philanthropy department provided a list of individuals who had expressed gratitude for their loved ones’ care within the Rush Program and who might have been amenable to participation. Finally, the principal investigator (JEF) presented Share the Care to leadership at CurePSP, a foundation dedicated to individuals with atypical parkinsonian conditions, including progressive supranuclear palsy, corticobasal syndrome, multiple system atrophy, and Lewy body dementia. As these diseases share many similarities with advanced PD, experienced caregivers from the CurePSP support group leader network were also eligible to participate as peer mentors and were referred directly by CurePSP leadership.

### Mentor and Mentee Eligibility Criteria

Peer mentors were recruited to provide emotional support to up to 2 mentees, sequentially, during the mentor program. All mentors were current or former caregivers who had at least 2 years of informal caregiving experience for an individual with PD or an atypical parkinsonian condition. Mentors were at least 30 years old and primarily English-speaking. They were required to attend a 1-time, in-person mentor training session at Rush University and commit to up to 2, sequential, 16-week blocks of peer mentoring (see Textbox 1 for a complete list of inclusion and exclusion criteria).

**Share the Care mentor and mentee eligibility criteria.**

**Mentor inclusion criteria**

- Aged ≥30 years
- >2 years of informal caregiving experience for an individual with Parkinson disease (PD) or a related disorder: dementia with Lewy bodies, progressive supranuclear palsy, multiple system atrophy, and corticobasal syndrome
- Previously participated in a caregiver support group for PD or related disorder, participated in a PD educational or outreach event, or given permission to be contacted for research
- English as primary language
- Able to attend a 5-hour mentor training session at Rush University
- Willing to commit to two, 16-week blocks of peer mentoring either in person, by telephone, or by videoconference for a minimum of 30 minutes per week
- Working telephone number

**Mentee inclusion criteria**

- Aged ≥30 years
- Caregiver in the Interdisciplinary Home Visits for Parkinson Disease Patient trial
- Cohabitating or spending ≥20 hours per week engaged in care-related tasks for a homebound individual with advanced Parkinson disease
- English as primary language
- Capacity to consent
- Working telephone number

**Mentor and mentee exclusion criteria**

- Terminal illness with a life expectancy of <12 months by self-report
- Exhibiting symptoms of a severe psychiatric disorder

All mentees were unpaid, informal caregivers for a homebound individual with advanced PD who had consented to and enrolled in the IN-HOME-PD study, the details of which have been described in detail elsewhere [39]. Mentees either cohabitated with or spent an average of 20 or more hours per week engaged in care-related tasks for a homebound, community-dwelling individual with advanced PD. If mentees were informal caregivers who subsequently obtained compensation for less than 1 quarter of their caregiving hours via local or state resources, these caregivers could participate. Mentees who self-reported a life expectancy of less than 1 year or who were exhibiting symptoms of a severe psychiatric disorder were excluded from participation. Mentees were matched with a mentor at the second of 4 quarterly home visits. All mentees completed the mentor program during the 16-week period between the second and third home visits.

### Intervention

#### Mentor Curriculum

**Figure 2:**
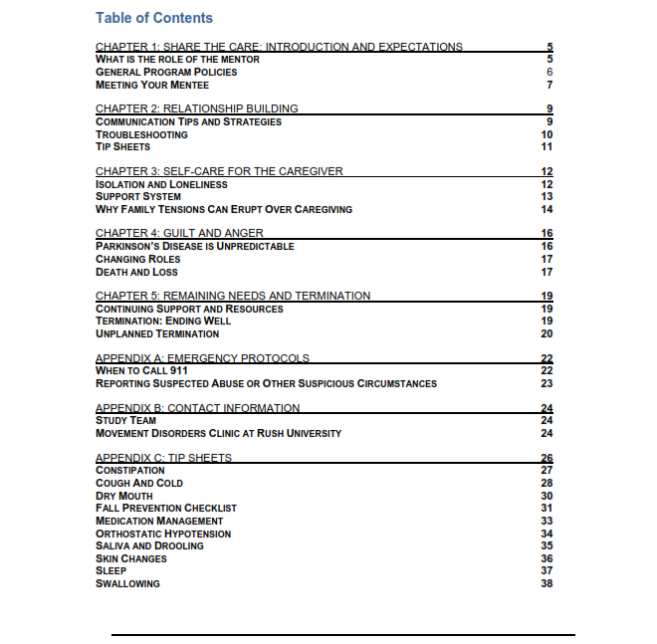
Handbook table of contents.

To provide a formal structure for the mentor program, we created the “Share the Care” mentoring handbook (table of contents shown in Figure 2; entire handbook in Multimedia Appendix 1). This handbook draws on topics identified as being important to individuals with advanced PD and their loved ones [41]. The handbook suggests key discussion topics for mentoring calls and guides mentors through the logistics, skills, and topics relevant to mentoring relationships.

#### Mentor Training

Potential mentors were provided with detailed information about the study and were screened for eligibility by phone by a member of the study team (ECK). Eligible individuals who elected to participate were scheduled to attend 1 of 3 five-hour, in-person training sessions at Rush University Medical Center. At the training session, mentors completed the informed consent process individually prior to any study activities taking place. After consent was obtained, mentors completed demographic questionnaires and baseline assessments of anxiety, depression, and caregiver self-efficacy (see Outcomes Measure below). Because all enrolled mentors had varying backgrounds and experiences with patients with PD and related disorders, a movement disorders specialist (JF) gave a detailed presentation on the rationale for the study, including prior home visit studies and research on PD caregivers. Next, lectures continued on the stages, common motor and nonmotor symptoms, and the management of sudden and gradual changes in advanced PD. In this way, all mentors were provided with a primer on advanced PD in order to relate to their mentees’ challenges, even if their own loved ones had not experienced certain symptoms. The study team’s social worker (JL) discussed the role of a mentor and mentorship skills, including being present, empathic listening, setting boundaries, maintaining confidentiality of all discussions, and brainstorming solutions to potentially challenging scenarios (eg, unrealistic mentee expectations of the mentor, unequal commitment to the study between the pairs, health crises, and life changes). The study team provided mentors with and reviewed the Share the Care handbook, offering suggestions on how to use it to support mentoring calls. All mentors were given the option of using a data plan–enabled iPad (Apple) provided by the study team to communicate with their mentees by videoconference. Mentors who elected to communicate this way were trained to use an iPad by a study team member (SPH, ECK, or JL). Mentors who preferred to use their own devices for videoconferences or who preferred to use telephone calls were permitted to do so.

#### Peer Mentoring

Given the rolling enrollment in the broader IN-HOME-PD study, each mentor was matched with 1-2 mentees over the course of the year following mentor training. To the extent possible, mentors and mentees were matched on the basis of similar age, sex, and relationship to a loved one with PD (eg, adult child, spouse or partner) although extensive matching criteria have not improved outcomes in prior peer mentoring work in AD/ADRD [28,42]. Each mentoring relationship lasted 16 weeks. Mentors were asked to speak with their mentee for 30 minutes weekly, either by phone, videoconference, or in person. Mentors were given their mentees’ contact information and asked to initiate mentoring. The mentors could refer to the “Share the Care” handbook to guide the weekly discussions but were free to focus on topics of concern to their mentees as they arose. Following the 16-week relationship with their first mentee, mentors had a break of 0-16 weeks, depending on their own scheduling needs and how many mentees were eligible to start mentoring at any given time. If available and necessary, mentors were then paired with a second mentee with whom they completed a second 16-week relationship.

Mentors and mentees maintained simple study diaries documenting when each conversation took place, how the pair connected (phone, videoconference, or in person), the length of the conversation, general topics discussed, whether they found the discussion useful, and any issues that occurred. To ensure fidelity, individual team members checked in with each participant at weeks 2, 4, 8, 12, and 16, when participants relayed the information in their study diaries to the study team member.

Finally, mentors were invited to participate in optional quarterly conference calls with other mentors and a member of the study team (JL). During these calls, mentors were invited to share their experiences and successes in their mentoring relationships, and to discuss any issues they had encountered. These calls provided opportunities for peers and the study team to validate the mentors’ successes and challenges, and for the study team to proactively address any concerns.

### Retention Efforts

Mentors received quarterly e-newsletters about the study and annual holiday cards to promote engagement and study retention. Upon study completion, the team sent each mentor a personalized thank you note and a certificate of appreciation. Mentee retention efforts included encouragement at quarterly home visits and interim follow-up calls regarding clinical care and psychosocial needs. In the event of a patient death, the study team sent a handwritten condolence card to the mentee and notified the respective mentor.

### Outcome Measures

Table 1 lists the instruments used to collect data from mentees and mentors, respectively, at each relevant time point during the study. For mentors, all baseline data were collected at the mentor training session. For mentees, demographic data were collected at the first home visit. Outcome measures were collected at home visit 1 (baseline), 16 weeks later at home visit 2 or week 0 of mentoring (prementoring baseline), 16 weeks later at home visit 3 (postmentoring), and finally 16 weeks later at home visit 4 (longitudinal follow-up).

**Table 1:** Assessments completed by mentees and peer mentors.

Domains		Instruments	Study visit							
			HV^a^ 1	HV 2	Wk 2	Wk^b^ 4	Wk 8	Wk 12	HV 3 Wk 16	HV 4
**Mentee**										
	Demographics	Demographics questionnaire	✓							
	Anxiety and depression	Hospital Anxiety and Depression Scale	✓	✓					✓	✓
	Self-efficacy	Caregiver Self-Efficacy Scale	✓	✓					✓	✓
	Caregiver strain	Multidimensional Caregiver Strain Index	✓	✓					✓	✓
	Study fidelity	Study diary – frequency, date, duration, topic of mentoring call			✓	✓	✓	✓	✓	
	Satisfaction with program	Client Satisfaction Inventory-Short Form							✓	
**Peer mentor**										
	Demographics	Demographics questionnaire	✓							N/A^c^
	Anxiety and depression	Hospital Anxiety and Depression Scale	✓						✓	N/A
	Self-efficacy	Caregiver Self-Efficacy Scale	✓						✓	N/A
	Study fidelity	Study diary: frequency, date, duration, topic of mentoring call			✓	✓	✓	✓	✓	N/A
	Satisfaction with program	Client Satisfaction Inventory Short Form							✓	N/A

^a^HV: home visit.

^b^Wk: Week of Share the Care mentoring program.

^c^N/A: not applicable.

### Mentee and Mentor Demographic Characteristics

Participants indicated their age, sex, race, ethnicity, highest level of education, primary language, marital status, relationship to care recipient with PD (or in the case of mentors, relationship to care recipient with PD or a related disorder), and years spent caregiving for that individual. As mentors were eligible to participate whether they were still actively caregiving or not, mentors alone were asked whether their care recipient was still alive. If living, the mentor was asked to disclose the care recipient’s living situation (eg, own home, assisted living facility, subacute or skilled nursing facility). If deceased, the mentor was asked to disclose how long ago the care recipient had died.

### Implementation: Fidelity and Satisfaction Measures

To examine fidelity to the Share the Care intervention, mentors and mentees were asked to complete study diaries to document when each conversation took place, the format (eg, phone call, videoconference), duration in minutes, topics discussed, whether or not they found the discussion useful, and any issues that occurred. Both mentors and mentees received check-in phone calls from study team members (ECK and JL) at weeks 2, 4, 8, 12, and 16. During these phone calls, mentors and mentees were asked to provide the information recorded in their study diaries; in the event that the participant had not documented an entry, the study team member asked them to provide a verbal response to each question. Finally, both mentors and mentees completed the Client Satisfaction Inventory Short Form (CSI-SF) [43] assessment at home visit 3 or postmentoring to measure their satisfaction with Share the Care.

### Primary and Secondary Outcome Measures

Among mentees and mentors, we assessed mental health using the HADS, a validated measure with individual anxiety and depression domains, for which scores >8 on either domain indicate probable symptoms [40]. Given the use of the HADS in prior peer mentoring interventions, this was selected as our primary outcome to facilitate sample size calculations and comparison with historical data [33]. As secondary outcomes, we administered the 9-item Caregiver Self-Efficacy Scale to both mentors and mentees at their respective baselines and at the end of the 16 weeks of mentoring [44]. This scale measures one’s belief in one’s ability to succeed in specific situations or accomplish a task, with domain subscores for symptom management and community support service use self-efficacy. Participants were instructed to complete this instrument according to how they felt on the day of administration; in the case of mentors who were no longer actively caregiving, they were prompted to answer as if they were still actively caregiving. As an additional secondary outcome, mentees completed the Multidimensional Caregiver Strain Index (MCSI) at home visits 1 and 2, and again after 16 weeks of mentoring. The MCSI is an 18-item assessment, validated in PD caregiver populations, spanning 6 dimensions of caregiver strain, with a score range of 0 (no strain) to 72 (worst possible strain) [3,45]. MCSI dimensions include physical, financial, and interpersonal strain; social and time constraints; and demanding behaviors on the part of the care recipient.

### Statistical Analyses

#### Sample Size Calculations

Sixty-five patient-caregiver dyads were enrolled in the larger IN-HOME-PD study based on a power calculation for the overarching study’s primary outcome of patient quality of life. All 65 caregivers consented to participate in the peer mentoring program as mentees. Assuming a mean HADS of 12 (SD 7) based on a trial of peer mentoring for caregivers of patients with dementia, 4 clusters of 15 caregivers each, a coefficient of variation of 0.3, a 2-sided significance level of 0.05, and power of 0.8, we concluded that 65 caregivers would afford the ability to detect a difference of 3.5 points in the HADS [30,33]. We assumed that each mentor would be paired with up to 2 sequential mentees, such that 34 mentors were recruited, assuming 10% attrition during the year-long mentor program.

#### Analytic Plan

We will use an intention-to-treat approach for all analyses, with per-protocol sensitivity analyses. We will describe the demographics, baseline depression and anxiety (HADS subscales), and self-efficacy of mentees and mentors, and caregiver strain (MCSI) of mentees only. To assess the implementation of the intervention, we will present the frequency and duration of mentoring calls. In the event of discrepant reports between the paired mentor and mentee, the number of calls or duration in minutes will be averaged. We will report the satisfaction of both mentors and mentees with the intervention using the CSI-SF and the percentage of calls rated as useful. Assessing for normality and using parametric or nonparametric tests as appropriate, we will compare within-subject change in anxiety, depression, and self-efficacy, respectively, over the 16 weeks of mentoring. We will then assess within-subject change of mentees and mentors each over the entire study for each of the primary and secondary outcomes to assess for crossover effects from home visits only (home visit 1 to home visit 2), Share the Care only (home visit 2 to home Visit 3), and the combined interventions (home visit 1 to home visit 4). We will construct linear regression models with change in depression, anxiety, strain, and self-efficacy, respectively, as the dependent variables, and a 16-week time frame (visits prementoring, visits with mentoring, visits alone postmentoring) or full study duration as the primary independent variable. We will adjust for potential confounders: caregiver demographics, mentoring visit frequency and duration, and risk factors for caregiver strain (motor fluctuations, falls, cognitive impairment, hallucinations, and poor quality of life) [3,8]. We will explore the heterogeneity of treatment effects using within-cluster comparisons of exposed (mentored) and unexposed (home visits only) time [46].

#### Data Management

Data were collected on paper case report forms and entered into a secure, regulation-compliant electronic database [47,48], with quarterly audits for fidelity. Data will be exported to Stata 15 (StataCorp) for analysis.

### Ethical Considerations

Approval was obtained from Rush University Medical Center’s Institutional Review Board on October 24, 2017 (number 17080209-IRB01). Two separate informed consent documents were developed for peer mentors and caregiver or mentees, respectively, including the details of their involvement in the study based on the 2 unique roles. All participants in the study provided written informed consent.

## Results

### Trial Status

Study recruitment began in February 2018 for mentees and May 2018 for mentors. We enrolled 65 mentees and 34 mentors into the program, and all mentors completed 1 of 3 in-person training sessions held by the study team in August 2018, November 2018, and February 2019. Following this, 33 mentors were matched with at least 1 mentee and all mentoring relationships concluded in November 2020. Due to pandemic-related delays, data cleaning and analysis were conducted throughout 2021, with dissemination of results anticipated to occur in the second half of 2022 via peer-reviewed publications and ClinicalTrials.gov.

### Baseline Characteristics of Mentors and Mentees

Baseline characteristics of mentors and mentees are shown in Tables 2 and 3. The majority of mentors (20/34, 59%) and mentees (51/65, 78%) were female. The mean age of mentors was 63.6 (SD 13.3) years and that for mentees was 66.1 (SD 6.4) years. Most participants identified as White (mentors: 26/34, 76%; mentees: 46/65, 71%) and were the spouse or partner of the person with PD for whom they were caring (mentors: 22/34, 65%; mentees: 39/63, 62%). Mentors enrolled in the program were all experienced caregivers, with an average of 8.7 (SD 6.4) years of caregiving experience. Only 4 mentors were still actively caring for their care recipient, while the remaining mentors’ care recipients were deceased.

**Table 2:** Baseline characteristics of caregiver peer mentors.

Characteristic			Outcome (N=34)
Age (years), mean (SD)			63.6 (13.3)
Female, n (%)			20 (59)
**Race, n (%)**			
	White	26 (76)	
	Asian	5 (15)	
	Hispanic	1 (3)	
	More than 1 race	1 (3)	
	Unknown/declined to answer	1 (3)	
**Care recipients’ diagnosis, n (%)**			
	Parkinson disease without dementia	10 (29)	
	Parkinson disease with dementia/Lewy body dementia	5 (15)	
	Multiple system atrophy	2 (6)	
	Progressive supranuclear palsy	16 (47)	
	Corticobasal syndrome	1 (3)	
**Relationship to care recipient, n (%)**			
	Spouse/partner/significant other	22 (65)	
	Adult child	11 (32)	
	Family friend or neighbor	1 (3)	
Caregiving time (years), mean (SD)^a^			8.7 (6.4)
Care recipient alive, n (%)			4 (12)
**Time since care recipient death (years), n (%)^b^**			
	Less than 1 year	5 (17)	
	1-2 years	9 (30)	
	2-5 years	14 (47)	
	More than 10 years	2 (7)	

^a^n=33.

^b^n=30; the remaining 4 mentors were still actively caregiving.

**Table 3:** Baseline characteristics of mentees.

Characteristic			Outcome (N=65)
Age (years), mean (SD)^a^			66.1 (11.5)
Female, n (%)			51 (78)
**Race, n (%)**			
	Caucasian	46 (71)	
	African American/Black	11 (17)	
	Asian	6 (9)	
	Unknown/declined to answer	2 (3)	
Hispanic, White, or declined to identify race, n (%)			4 (6)
**Care recipients’ diagnosis, n (%)**			
	Parkinson disease without dementia	40 (62)	
	Parkinson disease with dementia/Lewy body dementia	25 (39)	
**Relationship to care recipient, n (%)^b^**			
	Spouse/partner/significant other	39 (62)	
	Adult child	19 (30)	
	Other family member	3 (5)	
	Family friend or neighbor	4 (6)	
	Part-time home health aide	1 (2)	

^a^n=63.

^b^Mentors selected all relationships that applied.

## Discussion

This is the first structured study of peer mentoring for caregivers of homebound individuals with advanced PD, and to our knowledge, the first study of caregiver peer mentoring in PD in general. We expect that this novel pilot intervention will be met with high fidelity and satisfaction, given the unmet needs of this population of caregivers and the opportunity to share experiences with a knowledgeable peer. Caregiving in PD is associated with higher direct and indirect caregiving costs, greater strain and burden, and larger ramifications for caregiver health outcomes compared with caregivers of individuals with AD/ADRD [13,15,49,50]. A recent cross-sectional study confirmed that many PD caregivers rely on peers for advice [51], and a pilot study demonstrated the feasibility and high satisfaction of a caregiver tele-support group [52]. However, many behavioral interventions in PD have targeted the patient-caregiver dyad [53] or have been patient-focused with caregiver assessments as secondary outcomes [54]. For interventions aimed at improving caregiver outcomes, it may be necessary to limit activities to caregivers only or to provide opportunities for caregivers to participate apart from their care recipient. In the presence of the care recipient, social desirability bias and an understandable wish to preserve the care recipient’s privacy and dignity may limit the caregiver’s participation. Although Share the Care was nested within a broader patient-facing intervention, a particular strength is that the peer mentoring activities were entirely limited to the caregivers and their trained mentors, creating a safe and confidential space for discussing caregiving challenges, successes, and resources.

Several limitations arose in this pilot study. First, identifying and recruiting experienced mentors proved more difficult than had been anticipated. When individuals with PD have been institutionalized or have died, their caregivers may no longer be connected to the care recipients’ health care providers or social networks. This can pose a challenge both to recruitment from caregiver-facing sources, such as support groups or educational symposia, but also from provider referrals. Providers may be primed to recall caregivers of patients seen more recently rather than patients who have died or who have become estranged from routine care. We also found many individuals known to be deceased by their treating provider who were not marked accordingly in the EMR and adapted recruitment strategies to include provider reviews and clear descriptions of the time commitment required of mentors, as many had their own comorbidities or were still actively caregiving, which precluded the time necessary for training and mentoring. Due to the challenges recruiting PD caregivers as mentors, we expanded our recruitment to include mentors who had cared for loved ones with atypical parkinsonian disorders. We aimed to address the heterogeneity of mentors’ caregiving experiences through mentor training, acknowledging both that symptoms of advanced PD and the atypical disorders overlap significantly, and that even within PD, symptoms can vary from person to person. Nonetheless, the variability of mentors’ experiences may bias their interactions and limit generalizability. Furthermore, Share the Care was nested within the broader home visit study. Many caregivers consented to the entire study with the primary draw being home visits. For some caregivers, Share the Care might have been of lesser interest.

In future work, a peer mentoring intervention distinct from direct patient care may attract caregivers who recognize the need for additional support and who are explicitly interested in a peer mentoring relationship. Additionally, future directions include expanding eligibility to caregivers of nonhomebound individuals and offering virtual mentor training sessions to reach a larger and potentially national pool of mentors. Future iterations would be well-informed to incorporate behavior change theories and proven behavior change techniques to address frequently encountered complications in advanced PD and in caregiving for this population. Further studies are also needed to compare the efficacy and acceptability of individual mentoring with more traditional support groups or other group interventions.

Analysis of the Share the Care peer mentoring pilot study and the overarching home visit study is ongoing. We anticipate that the results and qualitative feedback from participants will inform the development of much-needed support interventions in families living with advanced PD and subsequently, across the disease spectrum. If successful, subsequent stand-alone programs will be developed and tested for caregivers of individuals with PD, atypical parkinsonian disorders, and AD/ADRD. Such a program could potentially impact the lives of millions of caregivers by providing information and resources while fostering connections with informed, experienced, and sympathetic peers. If successful, this model may also promote the transition of mentees into eventual peer mentors, building a pipeline of support for future caregivers. Given the rising prevalence of PD and the increasing reliance on family caregivers, effective and sustainable interventions are urgently needed to fill this critical gap.

## Appendix

Multimedia Appendix 1 Handbook distributed to caregivers.

## Abbreviations

AD/ADRD: Alzheimer disease and Alzheimer disease–related dementias
CSI-SF: Client Satisfaction Inventory Short Form
EMR: electronic medical record
HADS: Hospital Anxiety and Depression Scale
IN-HOME-PD: Interdisciplinary Home Visits for Individuals with Advanced PD
MCSI: Multidimensional Caregiver Strain Index
PD: Parkinson disease

## Data Availability

The deidentified data sets generated and analyzed during this trial will be made available from the corresponding author upon reasonable request.

## References

[1] Dorsey ER, Constantinescu R, Thompson JP, Biglan KM, Holloway RG, Kieburtz K, Marshall FJ, Ravina BM, Schifitto G, Siderowf A, Tanner CM (2007). Projected number of people with Parkinson disease in the most populous nations, 2005 through 2030. Neurology.

[2] Dorsey ER, Sherer T, Okun MS, Bloem BR (2018). The emerging evidence of the Parkinson pandemic. J Parkinsons Dis.

[3] Oguh O, Kwasny M, Carter J, Stell B, Simuni T (2013). Caregiver strain in Parkinson's disease: national Parkinson Foundation Quality Initiative study. Parkinsonism Relat Disord.

[4] Hely MA, Morris JG, Reid WG, Trafficante R (2005). Sydney Multicenter Study of Parkinson's disease: non-L-dopa-responsive problems dominate at 15 years. Mov Disord.

[5] Barone P, Antonini A, Colosimo C, Marconi R, Morgante L, Avarello TP, Bottacchi E, Cannas A, Ceravolo G, Ceravolo R, Cicarelli G, Gaglio RM, Giglia RM, Iemolo F, Manfredi M, Meco G, Nicoletti A, Pederzoli M, Petrone A, Pisani A, Pontieri FE, Quatrale R, Ramat S, Scala R, Volpe G, Zappulla S, Bentivoglio AR, Stocchi F, Trianni G, Dotto PD, PRIAMO study group (2009). The PRIAMO study: A multicenter assessment of nonmotor symptoms and their impact on quality of life in Parkinson's disease. Mov Disord.

[6] Fabbri M, Kauppila LA, Ferreira JJ, Rascol O (2020). Challenges and perspectives in the management of late-stage Parkinson's disease. J Parkinsons Dis.

[7] Spears CC, Besharat A, Monari EH, Martinez-Ramirez D, Almeida L, Armstrong MJ (2019). Causes and outcomes of hospitalization in Lewy body dementia: a retrospective cohort study. Parkinsonism Relat Disord.

[8] Abendroth M, Lutz BJ, Young ME (2012). Family caregivers' decision process to institutionalize persons with Parkinson's disease: a grounded theory study. Int J Nurs Stud.

[9] Hassan A, Wu SS, Schmidt P, Dai Y, Simuni T, Giladi N, Bloem BR, Malaty IA, Okun MS, NPF-QII Investigators (2013). High rates and the risk factors for emergency room visits and hospitalization in Parkinson's disease. Parkinsonism Relat Disord.

[10] Shahgholi L, De Jesus S, Wu SS, Pei Q, Hassan A, Armstrong MJ, Martinez-Ramirez D, Schmidt P, Okun MS (2017). Hospitalization and rehospitalization in Parkinson disease patients: data from the National Parkinson Foundation Centers of Excellence. PLoS One.

[11] Schrag A, Hovris A, Morley D, Quinn N, Jahanshahi M (2006). Caregiver-burden in parkinson's disease is closely associated with psychiatric symptoms, falls, and disability. Parkinsonism Relat Disord.

[12] Lau K, Au A (2011). Correlates of informal caregiver distress in Parkinson's disease: a meta-analysis. Clinical Gerontologist.

[13] Martinez-Martin P, Macaulay D, Jalundhwala YJ, Mu F, Ohashi E, Marshall T, Sail K (2019). The long-term direct and indirect economic burden among Parkinson's disease caregivers in the United States. Mov Disord.

[14] Martinez-Martin P, Rodriguez-Blazquez C, Forjaz MJ, Frades-Payo B, Agüera-Ortiz L, Weintraub D, Riesco A, Kurtis MM, Chaudhuri KR (2015). Neuropsychiatric symptoms and caregiver's burden in Parkinson's disease. Parkinsonism Relat Disord.

[15] Roland KP, Chappell NL (2019). Caregiver experiences across three neurodegenerative diseases: Alzheimer's, Parkinson's, and Parkinson's with dementia. J Aging Health.

[16] Hulshoff MJ, Book E, Dahodwala N, Tanner CM, Robertson C, Marras C (2021). Current knowledge on the evolution of care partner burden, needs, and coping in Parkinson's disease. Mov Disord Clin Pract.

[17] Vatter S, McDonald KR, Stanmore E, Clare L, Leroi I (2018). Multidimensional care burden in Parkinson-related dementia. J Geriatr Psychiatry Neurol.

[18] Vatter S, Stanmore E, Clare L, McDonald KR, McCormick SA, Leroi I (2020). Care burden and mental ill health in spouses of people with Parkinson disease dementia and Lewy body dementia. J Geriatr Psychiatry Neurol.

[19] Roth DL, Perkins M, Wadley VG, Temple EM, Haley WE (2009). Family caregiving and emotional strain: associations with quality of life in a large national sample of middle-aged and older adults. Qual Life Res.

[20] Butler M, Gaugler J, Talley K, Abdi H, Desai P, Duval S, Forte M, Nelson V, Ng W, Oullette J, Ratner E, Saha J, Shippee T, Wagner B, Wilt T, Yeshi L (2020). Care interventions for people living with dementia and their caregivers. Agency for Healthcare Research and Quality (AHRQ).

[21] Gaugler JE, Roth DL, Haley WE, Mittelman MS (2008). Can counseling and support reduce burden and depressive symptoms in caregivers of people with Alzheimer's disease during the transition to institutionalization? Results from the New York University caregiver intervention study. J Am Geriatr Soc.

[22] Fleisher J, Barbosa W, Sweeney MM, Oyler SE, Lemen AC, Fazl A, Ko M, Meisel T, Friede N, Dacpano G, Gilbert RM, Di Rocco A, Chodosh J (2018). Interdisciplinary home visits for individuals with advanced Parkinson's disease and related disorders. J Am Geriatr Soc.

[23] Fleisher JE, Sweeney MM, Oyler S, Meisel T, Friede N, Di Rocco A, Chodosh J (2019). Disease severity and quality of life in homebound people with advanced Parkinson disease. Neurol Clin Pract.

[24] Nwabuobi L, Barbosa W, Sweeney M, Oyler S, Meisel T, Di Rocco A, Chodosh J, Fleisher JE (2019). Sex-related differences in homebound advanced Parkinson's disease patients. Clin Interv Aging.

[25] Radder DLM, Nonnekes J, van Nimwegen M, Eggers C, Abbruzzese G, Alves G, Browner N, Chaudhuri KR, Ebersbach G, Ferreira JJ, Fleisher JE, Fletcher P, Frazzitta G, Giladi N, Guttman M, Iansek R, Khandhar S, Klucken J, Lafontaine A, Marras C, Nutt J, Okun MS, Parashos SA, Munneke M, Bloem BR (2020). Recommendations for the organization of multidisciplinary clinical care teams in Parkinson's disease. J Parkinsons Dis.

[26] Jennings LA, Reuben DB, Evertson LC, Serrano KS, Ercoli L, Grill J, Chodosh J, Tan Z, Wenger NS (2015). Unmet needs of caregivers of individuals referred to a dementia care program. J Am Geriatr Soc.

[27] Chen CK, Clayton K, Chodosh J (2017). The relationship between "What we believe" and "How we care" among daughters caring for a parent with dementia. Am J Alzheimers Dis Other Demen.

[28] Smith R, Greenwood N (2014). The impact of volunteer mentoring schemes on carers of people with dementia and volunteer mentors: a systematic review. Am J Alzheimers Dis Other Demen.

[29] Charlesworth G, Shepstone L, Wilson E, Reynolds S, Mugford M, Price D, Harvey I, Poland F (2008). Befriending carers of people with dementia: randomised controlled trial. BMJ.

[30] Charlesworth G, Burnell K, Crellin N, Hoare Z, Hoe J, Knapp M, Russell I, Wenborn J, Woods B, Orrell M (2016). Peer support and reminiscence therapy for people with dementia and their family carers: a factorial pragmatic randomised trial. J Neurol Neurosurg Psychiatry.

[31] Greenwood N, Habibi R, Mackenzie A, Drennan V, Easton N (2013). Peer Support for Carers. Am J Alzheimers Dis Other Demen.

[32] Greenwood N, Habibi R (2014). Carer mentoring: a mixed methods investigation of a carer mentoring service. Int J Nurs Stud.

[33] Charlesworth G, Sinclair JB, Brooks A, Sullivan T, Ahmad S, Poland F (2017). The impact of volunteering on the volunteer: findings from a peer support programme for family carers of people with dementia. Health Soc Care Community.

[34] Funnell MM (2010). Peer-based behavioural strategies to improve chronic disease self-management and clinical outcomes: evidence, logistics, evaluation considerations and needs for future research. Fam Pract.

[35] Bennett PN, St Clair Russell J, Atwal J, Brown L, Schiller B (2018). Patient-to-patient peer mentor support in dialysis: Improving the patient experience. Semin Dial.

[36] Perry E, Swartz J, Kelly G, Brown SL, Swartz RD (2003). Palliative care in chronic kidney disease: peer mentoring program personalizes advance directives discussions. Nephrol News Issues.

[37] Walshe C, Roberts D, Calman L, Appleton L, Croft R, Perez Algorta G, Skevington S, Lloyd-Williams M, Grande G (2021). Peer mentors for people with advanced cancer: lessons learnt from recruiting and training peer mentors for a feasibility randomized controlled trial. J Cancer Educ.

[38] Stevens Z, Barlow C, Iliffe S (2015). Promoting physical activity among older people in primary care using peer mentors. Prim Health Care Res Dev.

[39] Fleisher JE, Hess S, Sennott BJ, Myrick E, Wallace EK, Lee J, Sanghvi M, Woo K, Ouyang B, Wilkinson JR, Beck J, Johnson TJ, Hall DA, Chodosh J (2021). Longitudinal, interdisciplinary home visits versus usual care for homebound people with advanced Parkinson disease: protocol for a controlled trial. JMIR Res Protoc.

[40] Zigmond AS, Snaith RP (1983). The hospital anxiety and depression scale. Acta Psychiatr Scand.

[41] Fox S, Cashell A, Kernohan WG, Lynch M, McGlade C, O'Brien Tony, O'Sullivan Sean S, Foley MJ, Timmons S (2017). Palliative care for Parkinson's disease: Patient and carer's perspectives explored through qualitative interview. Palliat Med.

[42] Sabir M, Pillemer K, Suitor J, Patterson M (2003). Predictors of successful relationships in a peer support program for Alzheimer's caregivers. Am J Alzheimers Dis Other Demen.

[43] Mcmurtry SL, Hudson WW (2017). The client satisfaction inventory: results of an initial validation study. Research on Social Work Practice.

[44] Fortinsky RH, Kercher K, Burant CJ (2002). Measurement and correlates of family caregiver self-efficacy for managing dementia. Aging Ment Health.

[45] Stull D (1996). The multidimensional caregiver strain index (MCSI): Its measurement and structure. J Clin Geropsychology.

[46] Hemming K, Haines TP, Chilton PJ, Girling AJ, Lilford RJ (2015). The stepped wedge cluster randomised trial: rationale, design, analysis, and reporting. BMJ.

[47] Harris PA, Taylor R, Thielke R, Payne J, Gonzalez N, Conde JG (2009). Research electronic data capture (REDCap)–a metadata-driven methodology and workflow process for providing translational research informatics support. J Biomed Inform.

[48] Harris PA, Taylor R, Minor BL, Elliott V, Fernandez M, O'Neal L, McLeod L, Delacqua G, Delacqua F, Kirby J, Duda SN, REDCap Consortium (2019). The REDCap consortium: building an international community of software platform partners. J Biomed Inform.

[49] (2019). Caregiving for a person with Alzheimer's disease or a related dementia. Centers for Disease Control Alzheimer's Disease and Healthy Aging Program.

[50] Martinez-Martin P, Rodriguez-Blazquez C, Forjaz MJ, Frades-Payo B, Agüera-Ortiz Luis, Weintraub D, Riesco A, Kurtis MM, Chaudhuri KR (2015). Neuropsychiatric symptoms and caregiver's burden in Parkinson's disease. Parkinsonism Relat Disord.

[51] Mantri S, Edison B, Alzyoud L, Albert SM, Daeschler M, Kopil C, Marras C, Chahine LM (2021). Knowledge, responsibilities, and peer advice from care partners of patients with Parkinson disease psychosis. Front Neurol.

[52] Shah SP, Glenn GL, Hummel EM, Hamilton JM, Martine RR, Duda JE, Wilkinson JR (2015). Caregiver tele-support group for Parkinson's disease: A pilot study. Geriatr Nurs.

[53] Lyons KS, Zajack A, Greer M, Chaimov H, Dieckmann NF, Carter JH (2021). Benefits of a self-management program for the couple living with Parkinson's disease: a pilot study. J Appl Gerontol.

[54] Meinders MJ, Gentile G, Schrag AE, Konitsiotis S, Eggers C, Taba P, Lorenzl S, Odin P, Rosqvist K, Chaudhuri KR, Antonini A, Bloem BR, Groot MM (2021). Advance care planning and care coordination for people with Parkinson's disease and their family caregivers-study protocol for a multicentre, randomized controlled trial. Front Neurol.

