# Supplementary material for: Peer Mentoring Program for Informal Caregivers of Homebound Individuals With Advanced Parkinson Disease (Share the Care): Protocol for a Single-Center, Crossover Pilot Study
Source: JMIR Res Protoc. 2022 May 26;11(5):e34750. doi: 10.2196/34750 (PMC9185354; doi:10.2196/34750)
Supplement: Multimedia Appendix 1 [file resprot_v11i5e34750_app1.pdf]

SHARE THE CARE

Home Visit Mentoring Program Handbook

RUSH UNIVERSITY MEDICAL CENTER

SHARE THE CARE

Home Visit Mentoring Program Handbook

© Rush University Medical Center
1700 W Van Buren St • Suite 470
Chicago IL 60612

Table of Contents

CHAPTER 1: SHARE THE CARE: INTRODUCTION AND EXPECTATIONS 5

WHAT IS THE ROLE OF THE MENTOR 5
GENERAL PROGRAM POLICIES 6
MEETING YOUR MENTEE 7

CHAPTER 2: RELATIONSHIP BUILDING 9

COMMUNICATION TIPS AND STRATEGIES 9
TROUBLESHOOTING 10
TIP SHEETS 11

CHAPTER 3: SELF-CARE FOR THE CAREGIVIER 12

ISOLATION AND LONELINESS 12
SUPPORT SYSTEM 13
TENSION WITH FAMILY MEMBERS 14
WHY FAMILY TENSIONS CAN ERUPT OVER CAREGIVING 14

CHAPTER 4: GUILT AND ANGER 16

PARKINSON'S DISEASE IS UNPREDICTABLE 16
CHANGING ROLES 17
DEATH AND LOSS 17

CHAPTER 5: REMAINING NEEDS AND TERMINATION 19

CONTINUING SUPPORT AND RESOURCES 19
TERMINATION: ENDING WELL 19
UNPLANNED TERMINATION 20

APPENDIX A: EMERGENCY PROTOCOLS 22

WHEN TO CALL 911 22
REPORTING SUSPECTED ABUSE OR OTHER SUSPICIOUS CIRCUMSTANCES 23

APPENDIX B: CONTACT INFORMATION 24

STUDY TEAM 24
MOVEMENT DISORDERS CLINIC AT RUSH UNIVERSITY 24

APPENDIX C: TIP SHEETS 26

CONSTIPATION 27

Cough And Cold	28
Dry Mouth	30
Fall Prevention Checklist	31
Medication Management	33
Orthostatic Hypotension	34
Saliva and Drooling	35
Skin Changes	36
Sleep	37
Swallowing	38
When to Call 911	39

Chapter

1

Share the Care: Introduction and Expectations

SHare the Care is a caregiver mentoring program under the Home Visit Program (HVP). HVP provides interdisciplinary home visits for patients with Parkinson's Disease (PD) and their caregivers. Through the HVP, patients with PD are visited quarterly by a team consisting of a neurologist, nurse, social worker, and research coordinator. HVP has been shown to stabilize the quality of life of patients with PD. HVP has also brought attention to the immense strain caregivers feel as they care for their loved one with PD. Share the Care was conceived to address the caregiver's great need for support.

Past studies suggest that pairing current caregivers with former caregivers in a mentoring relationship provides benefits to both caregiver and mentor. Mentorship will offer you the opportunity to reflect on your life and perhaps grow as a communicator and role model. As a mentor, you may find it meaningful to share your stories and experiences of caregiving for a loved one with PD or a related movement disorder.

This handbook is meant to be a resource as you walk alongside your mentee. We are grateful for your participation, and hope that the Share the Care journey is a fulfilling one for you and your mentee.

What is the role of the mentor?

A mentor is someone who provides emotional encouragement and resource advocacy through a structured, personal relationship with their mentee. By "structured" we mean there is a specific start and end date, as well as formal program support and expectations. We believe the structure of Share the Care facilitates an open, reliable, and safe mentoring relationship.

As a mentor with Share the Care, your life experiences and the knowledge you've accumulated as a caregiver are invaluable to current caregivers. While this handbook specifies weekly topics of discussion, each mentoring relationship is unique and you should feel free to rely on your intuition as you build your relationship with your mentee. There may be some weeks where the assigned topic doesn't quite match your mentee's needs that week. Your mentee may appreciate simply having an experienced caregiver with whom to share fears, frustrations, and hopes. This handbook will discuss how to be an empathic presence in the life of your mentee, no matter the topic of the week.

You will become a trusted resource for current caregivers of loved ones with PD. However, you are not expected to be a miracle worker! You are not responsible for changing your mentee's situation or responding to their every request. Nor are you expected to act as your mentee's professional psychotherapist. In addition to your orientation session, you will be supported with biweekly calls from a study team member. You will also be asked to attend monthly supervision sessions with the study team. And of course, you should reach out to the study team anytime a concern arises.

General Program Policies

Time Commitment

Your time is valuable and we appreciate your generosity in meeting with mentees.

- You will receive program training through an orientation at Rush University Medical Center. The orientation session will provide an introduction to the Share the Care program goals, requirements, and practice in mentoring skills.

- Because Share the Care is part of the HVP study, you will regularly fill out surveys as well as diary cards that note your activities and feelings. The diary card is completed after each mentoring meeting, and a sample is included in the back of this book.

- You are committing to mentoring two separate mentees over a 12 month period. Each mentor-mentee relationship will last for 16 weeks. The two mentor-mentee relationships will have an approximately 16 week break between them.

- You will make at least one contact (which may be by telephone, in-person, or via iPad) with your mentee each week for the 16 week period, and fill out a diary card for each meeting. You may continue meeting with your mentee after the 16 week period, with the understanding that you will still be assigned a second mentee.

- A mentor-only monthly group supervision will provide ongoing training, as well as an opportunity for you to check in and share any concerns or successes you've experienced. The monthly meetings will take place via conference call.

- A member of the study team will check in by phone with each mentor and mentee every two weeks.

Confidentiality

It is the policy of HVP to protect the confidentiality of its participants and their families. With the exceptions below, information about mentors and mentees are protected under applicable law.

- *Safety*: When a person is at risk of being hurt, or hurting themselves or others, confidentiality may be breached as deemed necessary by study team members, mentors, and mentees. Mentors and mentees are expected to share information with study team members if there is ever a safety

issue or emergency. Further, both mentors and mentees should never hesitate to call 911 in an emergency. See more information about emergencies in Appendix B.

- *General Program Information*: For research purposes, study team members will review the study diaries of mentors and mentees. Mentors and mentees may also freely share any concerns regarding the mentoring relationship with research study staff.

Transportation

Mentors and mentees may transport each other in their private vehicles, if both parties agree. The driver assumes all costs and responsibility for such transportation.

Mentor-Mentee Visits

Mentors and mentees are encouraged to meet each week at a place and time that is mutually convenient. Mentor-mentee meetings may be in-person, by telephone call, or using the iPads provided through the study. Each pair is expected to meet for at least 30 minutes each week.

Meeting your Mentee

Now that you've been trained on the program and have had a chance to look through the Share the Care materials, it's time to meet your mentee!

Before your first meeting

Don't be surprised if you feel nervous as you think about your first meeting with your mentee. The first meeting is often the most nerve-wracking. You can prepare by going through this handbook, as well as reviewing the orientation materials. Your mentee may have questions about this program and how you can help. Through it all, remember that your mentee has been walking the hard road of caregiving – one that you know well! – and your simple presence and interest in your mentee is meaningful.

- The study team will have given you the name and contact information of your mentee, along with some background information. You should call your mentee soon after you receive this information and introduce yourself – who you are, your role as mentor for the Share the Care, and your interest in beginning the mentoring relationship.

- You and your mentee can decide on a time and place/method for your first meeting. It may be difficult, but it would be best to find a time that can work every week. Consistency can help avoid confusion and help reinforce the expectation that you and your mentee will be meeting weekly for the next 16 weeks.

During your first meeting

While this first meeting is a good time to learn more about your mentee, some mentees may not be ready to share too many details about their life. Below are some conversation starters for your first meeting. You are encouraged to first cover the logistics of Share the Care:

- The purpose of Share the Care is to provide weekly support to the mentee over 16 weeks. The hope is that your mentee will feel free to discuss with you difficulties that come up as a caregiver; questions about resources; or any successes or memorable moments of the past week.
- How will you and your mentee communicate between meetings, particularly if something comes up to prevent you from meeting as usual?
- How comfortable do you and your mentee feel talking over iPad or the telephone?

The first meeting is also a good time to share a little bit about yourself with your mentee:

- What made you interested in being a mentor?
- What are some highlights of your life?
- What do you like to do with your free time?

- If your mentee is open, you can also find out more about your mentee:
  - What were some jobs or hobbies that they had before becoming a caregiver?
  - What do they like to do now when they have time for themselves (or what do they wish they had more time to do)?
  - What are some of their expectations of the mentoring relationship?

At the close of this first meeting, confirm with your mentee your next meeting time and manner. You can express your appreciation for the mentor-mentee relationship, or affirm where your mentee is emotionally or physically.

After your first meeting

Congratulations on your first meeting with your mentee! No matter how you felt this meeting went, the time you gave and your willingness to walk alongside your mentee is much appreciated.

- It's a good idea to fill out your mentor diary card as soon as you can after your first meeting.
- Consider what you discussed, and if there's anything you want to bring up with study staff, whether immediately, during your biweekly call, or at your monthly check-in.

Chapter

2

Relationship Building

Suggested Weeks 1-4

DUring your first few meetings, which may be in person, via telephone, or via iPad, you and your mentee will be getting to know and trust each other. Your mentee may be looking to you to take the lead in initiating conversations.

Communication Tips and Strategies

Think back to a time that you felt particularly *understood* by a family member or friend. What was it about the way that person communicated that made you feel like they "got" you? Below are some characteristics of effective communicators.

- Non-judgmental stance. Effective communicators enter a conversation open to what the conversation will bring. Your personal experience as a caregiver is a great strength, but your mentee might be having an experience very different from yours. Be available to whatever your mentee is currently going through, and try to notice – and gently lay aside – whatever preformed ideas you may have about your mentee or his or her caregiving. Let your mentee tell his or her story completely before offering your thoughts.

- Active listening. Have you ever found yourself in a conversation where it felt like someone is just waiting to have his or her turn to speak? In *active listening*, we are not just forming a response in our head while someone is speaking. We are being attentive and responsive to the mentee's communications. You can nod your head along, give verbal affirmations like "I see" and "uh-huh" and summarize back to your mentee what you've heard, so that you both know you are on the same page.

- Open-ended questions. Close-ended questions can be answered with a simple "yes," "no," or other short responses. They are useful for focusing on specific issues. Open-ended questions, on the other hand, require more thought and are useful in encouraging your mentee to explore the problem or think out loud. Questions that begins with "why," "how," or "what" encourage reflection.

- Validation. During your mentoring relationship with your mentee, your mentee may say or do things with which you disagree. Though your initial reaction may be a strong one, try to always respond to and validate your mentee's emotions before addressing the underlying issues. There is no such thing as a "wrong" emotion, and your mentee may just need to hear that it's ok to feel the way he or she is feeling. For example, your mentee may be having an especially frustrating day and confess that they wish they could end the caregiving relationship. Rather than jump immediately into whether that's a good or bad idea, take some time to affirm the difficulty of caregiving and the burden your mentee has been carrying.
    - Examples of validating statements: "I can see why you feel that way"; "It must be painful and difficult to have something like that happen"; "That sounds discouraging"; "I can see this is important to you."

Troubleshooting

You may find, especially in the first few weeks of your mentoring relationship, that the conversation with your mentee doesn't flow very easily. Or you may feel frustrated if you and your mentee don't seem to be clicking, no matter how hard you try. It's difficult, but try not to take it personally! As you may have experienced, caregiving can be incredibly stressful. Even if your mentee does not express it, your simple interest in his or her life is supportive.

There may also be occasions where your interaction with your mentee brings you distress – perhaps your mentee has shared something for which you've felt unprepared, or perhaps your mentee's communications remind you of painful events. The HVP study team is always available to help process any interaction and to provide support. The hope is that your relationship with your mentee is not just one-sided; the hope is that walking alongside a current caregiver will also be a growing and fulfilling experience for you.

If you are finding it difficult to connect with your mentee, take a step back and ask yourself what your expectation of your mentee is; we all have unspoken expectations when we enter a relationship, and identifying these expectations can help us understand our frustrations. Would you have liked a mentee that was more engaged, or maybe even more outwardly appreciative of your time and effort? Perhaps your mentee has expressed that he or she doesn't see the need for a mentor.

It is a challenge to mentor when your mentee doesn't match your level of enthusiasm or engagement. There may even be some weeks where you don't quite feel enthusiastic about your interactions with your mentee. As much as you can, embrace these ups and downs of the mentoring relationship. You can be authentic about the challenges with your mentee, even acknowledging aloud that it seems both of you are feeling a little uncertain about the relationship. We also encourage you to remain positive about mentoring, voicing hope and encouragement about the mentoring process. We, as the study team, are here to walk alongside you as you mentor a caregiver, and we have confidence that the process of mentoring – including any difficulties – will be a rewarding one.

Tip Sheets

Each caregiver/mentee has a binder (the "Caregiver Binder") that includes "tip sheets" that offer practical information about different symptoms related to PD, including constipation, orthostatic hypotension, excess saliva, and other physical difficulties with which you may already be familiar. These

tip sheets are included at the end of this binder. The study team will go over the Caregiver Binder with the mentees, but your mentee may also have questions for you that can be answered from these tip sheets. Please review the tip sheets, and freely refer your mentee back to these tip sheets as appropriate. If you have questions about any information in the tip sheets, please do not hesitate to reach out to a study team member.

Keep in mind that, while you should share what has worked for you in caring for your loved one, you are not responsible for the medical health of your mentee or his or her loved one. If your mentee contacts you with a medical emergency, please encourage him or her to call their provider or 911 as appropriate. You can read more about emergency protocols in Appendix B.

Chapter

3

Self-Care for the Caregiver

Suggested Weeks 5-8

ONe of the most difficult parts of caregiving is finding time to take care of the self. Most of us can recall buckling into an airplane seat and having the stewardess tell us that, in an emergency situation, we should put the oxygen mask on ourselves before we try to help anyone else. Even though the same principle holds in caregiving, it's hard to prioritize ourselves when our loved one's needs are so pressing and visible.

In fact, ignoring our own needs as a caregiver might be a lifelong pattern. There are many reasons why some of us find it easier to take care of others than ourselves: we feel it's selfish to prioritize ourselves (and someone may even have said that directly to us); we may feel we are not a good partner or family member unless we sacrifice ourselves completely; or we may have a hard time admitting we need help.

In this chapter, we list many conversation starters and questions that may get your mentee thinking actively about self-care.

Isolation and Loneliness

Caring for someone who is homebound may be the hardest thing your mentee has ever done. You may remember the loss of having to give up your usual schedule and activities, and the difficulty of maintaining your social connections as more and more of your energy becomes focused on your loved one.

Often, emotional isolation accompanies physical isolation. Caregivers may not know anyone in a similar situation, and anger and resentment about their changed circumstances are normal. Caregivers may even feel angry at their friends and family for not doing more or showing more support, leading to more isolation. Social isolation has been proven to deteriorate emotional and physical health, and it ultimately makes it more difficult to be a caregiver. As a mentor, you can be a reminder that there are others who have also served as caregivers and understand the complicated emotions that arise.

Some questions you can ask to help your mentee reflect on their possible isolation are below. You don't need to ask all of them, but they may provide an opening to a good conversation about how your mentee's social activities have been impacted by caregiving. The communication tips and strategies from Chapter 2 may be handy as you engage in this discussion with your mentee.

- What are some specific ways in which your daily life has changed since you've started caregiving for your loved one?

- Who do you talk to regularly, outside of your loved one? How much time do you take to meet with friends or family members in a given week?

- What kind of physical activity do you currently engage in? What are some specific obstacles to engaging in this activity regularly?

- What do you do that has brought you joy in the past? Is there a way you could incorporate any element of those activities in your current life?

Support System

Your mentee may admit that their current daily life isn't sustainable in terms of their emotional and physical health. However, it may be difficult for your mentee to make changes, due to their guilt, their practical circumstances, or other obstacles.

As a mentor, you have become an important part of your mentee's support system. As you walk alongside your mentee, you can share what has helped you when you were caregiver: who or what provided meaningful support to you, what were some obstacles to accessing that support, and what were some creative ways you worked around those obstacles?

You can help your mentee identify additional individuals that can provide emotional and practical support by exploring the following questions with your mentee.

- What are your relationships like with other family members? How much do other family members know about your current situation, and how comfortable are you sharing with family members? If you don't feel comfortable, why not?

- Are you a part of any community group, such as a faith community, a senior citizens center, support group, or other social group? Why or why not?

- Is there a social worker working with you? If so, how aware is the social worker of your caregiving challenges?

- Have you noticed any signs of depression and anxiety in yourself, such as losing interest in things that once were pleasurable, feeling fatigued and low-energy, and feeling a sense of hopelessness? If so, have you considered professional help?

And, of course, don't be shy about drawing from your own experiences in ways not covered above. You are not responsible for providing a solution to your mentee's situation, but you can be a sounding board and gentle partner in discussing these issues.

Tension with Family Members

PD is a progressive disease, and the decisions surrounding the care of a loved one with PD may get more difficult as time passes. Family members can be a source of invaluable comfort and support in the caregiving process, but family members can also be a source of conflict in this stressful time.

While a social worker can help your mentee with practical issues like planning a family meeting or coordinating caregiving with relatives, you can play an important role in exploring the challenges your mentee may be having with family members.

Why Family Tensions Can Erupt Over Caregiving

Older adults may be living longer than ever, but it is often with a chronic condition. This means that children can end up caring for their parents for decades. Making joint decisions on what is best for the parent can bring back feelings and associations from childhood – the same patterns of interaction from childhood might show up now as adults. It's not uncommon for siblings to have conflict over how much financial or physical support is being given for the parent; where the parent is living; and how to best treat the parent's ailments. Below are some strategies you may share with your mentee if he or she is navigating sibling or other family relationships in the process of caregiving. These tips are adapted from https://www.caregiver.org/caregiving-with-your-siblings.

- <u>Be open and willing</u>. Your mentee's relatives may be making decisions or saying things your mentee doesn't agree with, but the more your mentee shows a willingness to consider that his or her relatives might have a good point, the more likely they will be willing to work with your mentee. Your mentee can show openness and willingness by letting others have their say without interrupting, and finding one or two good things to say about others' ideas before critiquing the ideas.

- <u>Avoiding "always" and "never" statements</u>. In moments of stress, it's easy to oversimplify people and conclude that they "never" do something or are "always" a certain way. Specificity works better than generalizations – referring to a specific instance or interaction that was troubling is more effective than all-or-nothing statements that distract from what your mentee really wants to communicate.

- <u>Your mentee should ask him or herself what's really desired from relatives</u>. Many caregivers feel overburdened and stretched thin by their responsibilities but, when offered help, decline it. Sometimes, it's more of a hassle to have someone else involved in the day-to-day caregiving – it might require letting someone else into their space, or sharing control with someone they don't entirely trust. It's important that your mentee be as honest as possible about what would truly be helpful. Is it money; emotional support; or occasional time off? Perhaps it's simply heartfelt and regular acknowledgement of the mentee's hard work in caregiving.

- <u>Make detailed and realistic requests.</u> Many of us are not accustomed to directly asking for help. We send hints, or think "we shouldn't have to ask; they should just know." Unfortunately, this stance often leads to conflict. Once your mentee knows how relatives can best help in the caregiving, the best communication is the most specific one: asking if a sister can sit with the loved one every Thursday morning, for example, or if a brother can send a specific amount of

money for groceries at the end of each month. Additionally, your mentee's requests are more likely to be met if they are realistic. If your mentee's brother feels financially stretched thin, your mentee may have to think of other ways he can contribute.

- Watching tone and language. In making decisions around caregiving, there are many opportunities for miscommunication. Sometimes – especially when we're stressed – our tone and language may come across more negative than we intend. This is true of others as well. Encourage your mentee, when in doubt, to give the benefit of doubt and assume that others have the best intentions, even if their tone and language is off-putting. On the flipside, you might suggest to your mentee that his or her requests and critiques are more likely to be heard if they are made with an easy tone and language.

In the day-to-day stress of caregiving, it may be easy for your mentee to lose sight of the fact that family relationships will likely endure beyond the end of the caregiving role. Compassion for oneself as well as for one's family members as they journey the hard road of caregiving can go a long way in defusing potential family conflict.

Chapter

4

Guilt and Anger

Suggested Weeks 9-12

THere is no shortage of guilt in caregiving. Loved ones may feel guilty because of the disruption and burden their disease causes caregivers. Caregivers may feel guilty over the limits of their caregiving, or difficult decisions that cause pain to the loved one. Guilt makes us feel uncomfortable and vulnerable. Often, we react to these feelings of discomfort and vulnerability by lashing out in anger, which leads to more guilt.

Your mentee may be caught in this cycle of guilt and anger without realizing it. Below, we discuss common occasions for staying in this cycle, and how you can help your mentee see how he or she might be stuck.

Parkinson's Disease is Unpredictable

One of the biggest frustrations for PD patients is the "on" and "off" phenomena: when their body is "on," symptoms are minimal and they can have a close to normal day. On "off" days, patients feel that they have lost control over their body, and their symptoms seem worse than ever. While medications can help regulate the "on" and "off" periods, the unpredictability of the disease can cause anxiety. Your mentee's loved one may not want to have social interactions because they aren't sure what their body will do. Your mentee may feel apprehensive, not knowing what kind of needs his or her loved one will have on a particular day.

PD is also unpredictable in its progression. Some individuals function for decades with the disease, while others face a fast decline. The unpredictable progression of PD means that no one roadmap will be appropriate for every patient. Your mentee may have made some promises in the past that are now difficult to keep. These promises may be about who will be providing care, or where the loved one will live. Changes to these promises can bring distress to both your mentee and his or her loved one.

As a mentor, your experience in this journey of uncertainty is invaluable. How did you come to live with your loved one's diagnosis and all the uncertainty that it entailed? You can validate your mentee's fears about the future – how frightening it can be to not know how much care their loved one will need or how they will manage to continue to provide that care. You can also help your mentee validate his or her loved one's fears. Your mentee's loved one may be fearful of losing ever more independence, or of becoming an unmanageable burden. You can encourage your mentee to acknowledge and affirm

these difficult emotions without making explicit promises about who will care for them or where they will live.

Changing Roles

As your mentee's loved one progresses, your mentee may find that he or she has switched roles with the loved one. Perhaps your mentee's loved one used to take care of the finances and household affairs but is now unable to. Or perhaps it was the loved one that arranged social activities and is now too anxious to go out.

No matter the specific nature of the changes, life is not what it used to be, either for your mentee or his or her loved one. These changes, like so many aspects of PD, can elicit anger, resentment, and frustration in your mentee. Your mentee may then feel guilty for having these "negative" emotions towards their loved one.

Every Emotion is Appropriate

It would be helpful to remind your mentee that there are no "wrong" emotions. We are sometimes fearful of the "negative" emotions we feel because it might mean there is something wrong with us or our relationship with our loved one. This leads us to hide those "wrong" emotions and not be honest about what we are feeling. *Feeling* angry or resentful is not wrong. It's what we do with those emotions that matters.

When we have strong emotions, we should find a safe person with whom we can be honest, and eventually find a way to be honest with our loved one. For your mentee, that safe person might be you, or some other person. Don't feel offended, as a mentor, if your mentee chooses not to completely share complicated emotions with you. That feeling of safety may come over time. In the meantime, you can validate all the emotions your mentee has and, if you're comfortable, share any complicated emotions you've experienced in your own journey with caregiving. Your mentee might find it reassuring to simply know that it's not abnormal to have a mix of emotions as roles change.

You can also point your mentee to the study team social worker to help manage the logistics of changing roles. Perhaps your mentee is struggling with keeping up with the bills, or feeling socially isolated because of his or her new role. The social worker will be able to locate resources that might address these issues.

Death and Loss

It is sadly possible that your mentee may experience the death of his or her loved one in the course of your mentoring relationship. Just as there is no "right" emotion in response to changing roles in PD, so there is no "right" emotion in response to death. The end of the caregiving role can leave a sense of emptiness or meaninglessness in the caregiver. At the same time, some caregivers and their loved ones also look to death as a relief from the cares that they carried. There is no script for reacting to death, and the most important factor in conversations about death is kindness and patience as your mentee works through what death might mean to him or her.

Grief refers to the physical, emotional, and behavioral reactions individuals have to the loss of a loved one. While the "five stages of grief" (denial, anger, bargaining, depression, and acceptance) has become

a popular concept, in reality each person experiences loss in unique ways and not all five stages apply to all people. That's ok. There is no correct way to grieve the death of a loved one. If your mentee feels overwhelmed by the emotions that come with loss, a professional therapist may be able to help him or her.

Mourning refers to ritualized expressions of grief, such as holding funerals or memorials, or wearing black colored clothing. Sometimes it's helpful to come up with special forms of mourning that reflect the uniqueness of the loved one who has passed away. Some people like to light candles, or release paper lanterns, or even have a special meal in honor of their loved one. If your mentee is struggling with expressing his or her emotions after loss, he or she might find comfort in performing a personal mourning ritual.

Self-Care for Yourself as a Mentor

On a final note, as a mentor, you may have had your own experience with loss, and your mentee's possible loss may bring up complicated emotions. You may face painful memories, or feel your mentee's emotions as intensely as if they were your own. Just as your mentee needs support in his or her grief, you also may need support in this difficult time. Don't hesitate to reach out to any of the study team members and let them know you would like to talk.

Chapter

5

Remaining Needs and Termination

Suggested Weeks 13-16

THese are the last few weeks of the mentoring program! You should feel proud of the growth that has occurred in your mentoring relationship. You will spend the next three weeks bringing closure to your relationship; this is a great opportunity to reflect with your mentee on the mentoring relationship, as well as share hopes for the future. You've worked hard on this mentoring relationship and we want to help you end on a strong note.

Continuing Support and Resources

Though your official role as a mentor is coming to an end, your mentee will likely continue on in his or her role as caregiver and may have ongoing needs for support and resources. You may have mixed feelings about ending the mentoring relationship if your mentee is going through a period of distress. Please do not hesitate to share any concerns you have with the study team. The study team will continue to work with your mentee and strive to address the ongoing needs of your mentee. You can encourage your mentee to reach out directly to the study team's social worker with any need or questions.

As a mentor, you will also have a termination session with the study team where you can look back on your time as a mentor and share feedback about the program. You have engaged in a significant 16-week mentoring relationship with an individual that had been a stranger to you prior to this program. It is normal to have a range of emotions at the end of this program and the study team will be available to you even after the termination session to provide support as you process your relationship with your mentee.

Termination: Ending Well

A good ending is a critical part of the mentoring process – it helps both you and your mentee achieve a sense of closure and acknowledges that each of you is transitioning into a different phase. Below are some tips on how you can end well with your mentee.

- The first step to ending well is to clearly establish the exact end of the mentoring relationship. You should give your mentee two or three weeks' advance notice of the date of your last meeting. Terminations are generally recommended to be in person, but do not feel badly if that's not possible.

- If you would like to keep in touch with your mentee, be specific about what your mentee can expect – would you want your mentee to occasionally call you, or would you prefer to be the one to occasionally call your mentee? Do not feel pressured to make any promises that may be difficult to keep; you should feel free to honestly and kindly state that you do not think you will be able to keep in touch after the mentoring program ends.

- At your very last session, you and your mentee should reflect on the difficulties, triumphs, and overall experience of the mentoring relationship. Examples of things you can share with your mentee are:

    - Your mentee's unique and special qualities that you appreciated.

    - Interactions that stand out, and what touched or moved you about your mentoring relationship.

    - What you will take away from your time with our mentee, and how it may have changed you.

    - What you want your mentee to take away from the relationship.

    - Hopes and wishes for your mentee, and what you will miss about your mentee.

    - Any parting advice in caregiving.

Unplanned Termination

The monthly check-ins with the study team are opportunities to identify difficulties in the mentoring relationship as they arise. Hopefully, you have felt safe seeking support in any tension, discomfort, or low engagement you have experienced in your mentoring relationship. However, there may be occasions when early termination, either by you or by your mentee, is unavoidable. You or your mentee may be physically unable to continue the relationship because of illness, relocation, or other personal circumstances.

If you think that you will no longer be able to participate in Share the Care, please let the study team know as soon as possible. The study team will work with you in informing your mentee and taking appropriate steps to end the mentoring program early and well.

If your mentee needs to terminate the mentoring relationship early, the study team will contact you as soon as possible to let you know. Further, the study team will work with you and your mentee to arrange for closure.

Thank you for your investment of time into Share the Care. Your participation has made a difference, not just for your mentee, but research on PD and movement disorders. The time you have spent with

your mentee, as well as the information you have shared with us, will help us improve the lives of PD patients and their caregivers.

Emergency Protocols

As a mentor, you can help point your mentee to the resources they need and offer emotional support. However, you are not responsible for your mentee's well-being. This is especially true in crisis situations. Your mentee may contact you in crisis – perhaps your mentee feels psychologically distressed, or his or her loved one has had a medical emergency. Below are important principles if your mentee calls you or otherwise lets you know that he or she is in a crisis:

- Use a calm voice and gently encourage your mentee to take some deep breaths – this will help your mentee to calm down.

- Ask your mentee to describe the situation or problem, and ask your mentee if he or she has sought help from anyone else yet.

When to Call 911

<u>If your mentee is describing any of the following situations, have your mentee call 911 immediately</u>. If your mentee is unable or unlikely to call 911, keep him or her on the line or in your presence as you locate another phone with which to call 911. If you must hang up on or leave your mentee, note your mentee's location and phone number before calling 911. When to call 911:

  - There is a crime, fire, or accident that is ongoing or just has happened;
  - Your mentee has disclosed that he or she has already attempted to harm him or herself, or someone else;
  - Your mentee or his or her loved one has had a meaningful medical incident including, but not limited to:
    - A fall;
    - A cut that results in uncontrollable bleeding;
    - Having chest pain;
    - Is gasping for air or not breathing;
    - Experiencing a severe allergic reaction;
    - Or any other significant symptoms.

- If you're not sure whether the situation is a true emergency, call 911 and let the call-taker determine whether you need emergency help.

- When you call 911, be prepared to answer the following questions:
    - The location of the emergency, including the street address;
    - The nature of the emergency;
    - Details about the emergency, such as a physical description of a person who may have committed a crime, a description of any fire that may be burning, or a description of injuries or symptoms being experienced by a person having a medical emergency.
- If you're not sure whether the situation is a true emergency, call 911 and let the call-taker determine whether you need emergency help.
- Let the study team know, at your earliest convenience, of the call to 911.

Reporting Suspected Abuse or Other Suspicious Circumstances

If, over the course of your mentoring relationship, you begin to suspect that your mentee or his or her loved one is in an unsafe situation at home, let the study team know as soon as possible. The study team staff will work with you to contact Adult Protection Services if necessary, or otherwise request a wellbeing check from senior service agencies in the mentee's area. If you think your mentee or loved one is in immediate danger, do not hesitate to call 911.

In general, call 911 with any immediate concerns about harm to self or others. If there are any circumstances in which you feel uncomfortable or sense that something is not right, let the study team know as soon as possible.

Contact Information

The study team will check in with you regularly to provide ongoing support, training, and assistance with any issues. You should also feel free to reach out to the study team at any time for any reason.

Study Team

Members of the study team are listed below. Please note that the study team may be out of the office on home visits and may not be immediately available to take your call. If you leave a voicemail, your call will be returned as soon as possible.

- Claire Niemet, Research Coordinator
  312-563-0676
  Claire_j_niemet@rush.edu

- Ellen Klostermann Wallace, Clinical Research Coordinator
  312-563-0674
  eckw@rush.edu

- Jeanette Lee, Licensed Social Worker
  312-942-4951
  Jeanette_lee@rush.edu

Movement Disorders Group at Rush University

Dr. Jori Fleisher, the neurologist that is treating all participants in the Home Visit Program, is a part of the Movement Disorders Group at Rush University. She, as well as other medical providers caring for your mentee's loved one, may be reached at the following:

Rush Movement Disorders Group
1725 W Harrison Street
Chicago, IL 60612
(312) 563-2030

Tip Sheets

The following tip sheets appear in your mentee's "Subject Binder" that he or she received from the study team at Visit 1 of the Home Visit Program. It may be helpful for you to be familiar with these tips sheets in case your mentee has questions about these common symptoms of PD.

TIP Sheet: Preventing and Managing Constipation

The best way to manage constipation is to prevent it.

Goal: Aim to have a bowel movement at least 1 time every other day.

Step 1:

Eat a **high fiber diet**

For example: Apples, carrots, oatmeal, bran, broccoli, dried fruits

Drink plenty of **fluids**

Exercise daily

Step 2:

Try: Prune Juice Cocktail: ½ cup applesauce, 2 tablespoons wheat bran and 4-6 ounces prune juice. Drink daily to prevent constipation.

OR

Rancho Recipe: 1 cup applesauce, 1 cup bran, and ¾ cup prune juice. Mix together until it is the consistency of peanut butter. Take two tablespoons daily with water, or mix with breakfast foods like smoothies or oatmeal. This should be kept refrigerated for up to one week.

Step 3: *If the above recipes do not work*

Over-the-counter medications:

Docusate sodium (Colace, Dulcolax) 100mg capsules
Take one capsule by mouth 1-2 times daily

OR

Polyethylene glycol (Miralax, Glycolax)
Dissolve one packet or 1 capful of powder in 4-8 ounces of water and drink 1-time daily

Notes: If you take bulk laxatives like Metamucil or Citrucel make sure you drink plenty of water (at least 8-16 ounces) otherwise your constipation could get worse.

TIP Sheet: Cough and Cold Medicines with Parkinson's Disease

There are many cough and cold medications sold under different brand names and in different combinations. Here is a description of the different types of medications and how they can interact with Parkinson's disease (PD) medications. There is a list of safe cough and cold medications, as well as a list of medications to avoid. If you are not sure if a product contains the mentioned drug, please ask your pharmacist and tell them what medications you take for PD. All the cough/cold medications should be taken according to the instructions.

Cough Medications

Dextromethorphan is a cough suppressant and common ingredient in many cough and cold medications, either by itself or in combination with other ingredients. Common brands that often contain dextromethorphan are the "DM" brands, like Robitussin DM, Mucinex DM, Delsym, Vicks, Theraflu, and Triaminic, as well as other generic store brands. People taking the MAO-B inhibitors selegiline (Eldepryl) or rasagiline (Azilect) for PD should not take dextromethorphan. Dextromethorphan may also interact with certain antidepressants, so check with the pharmacist if you are on anti-depressants and need to take a cough suppressant.

Guaifenesin is an expectorant cough medicine, which means it thins secretions and helps bring them up from the throat and lungs. Guaifenesin can be found in many cough and cold products, like Robitussin, Mucinex, Q-Tussin, and many other combination products. Guaifenesin is safe when combined with any of the available PD medications.

Decongestants

Oral decongestants are drugs found in cough and cold medicine for stuffy, runny nose. Drugs containing oral decongestants are often labeled "Day" or "PE" drugs. They include Pseudoephedrine and phenylephrine. These drugs are generally safe in combination with most PD drugs, but are best avoided with the MAO inhibitor selegiline (Zydis, Eldepryl) because of an increased risk of high blood pressure. Decongestants can be taken with rasagiline, which is a newer MAO inhibitor. So the labels of many oral decongestant drugs may say that they should not be avoided in PD, but this is not accurate: they should only be avoided in patients taking selegiline. So, if a decongestant is needed, it can be used in PD patients unless the patient is also on selegiline, or midodrine, a medication for low blood pressure.

Antihistamines

Many cold and allergy preparations contain antihistamines. They are often used for runny nose, sneezing, itchy or watery eyes, or allergic reactions like hives. Two of the anti-histamines are sedating and in older adults can cause confusion. These are diphenhydramine (Benadryl, Tylenol PM, Advil PM) and chlorpheniramine (Chlor-Trimeton). While these drugs can be used in people with PD, their use should be limited in older patients and those with confusion or excessive sedation in the past.

Other anti-histamines that are safe to use in PD include loratadine (Claritin) and cetirizine (Zyrtec).

Pain/Fever Reducers
Many cough and cold products contain ingredients for pain or fever. The most common products include acetaminophen (Tylenol), ibuprofen (Motrin or Advil), and naproxen (Aleve). These are considered safe medications that won't interact with PD medications.

Antibiotics
Most antibiotics are safe with PD medications, but rasagiline (Azilect) may interact with the antibiotic ciprofloxacin. Ideally, a different antibiotic would be prescribed; if this is not possible, rasagilne would need to be stopped temporarily while ciprofloxacin is being taken.

Notes: If you are not taking any prescribed medications for PD, then there are no restrictions on the cough and cold medications you can take.

TIP Sheet: Dry Mouth & Parkinson's Disease

While Parkinson's disease and Parkinson's disease medications can certainly cause dry mouth on their own, older age, mouth breathing, and anxiety can also contribute. Dry mouth is not just a discomfort but can lead to gum disease, dental cavities, and tooth loss.

Easy first-line strategies are:

- Increase fluid intake (water, other sugar-free decaffeinated beverages)
- Rinse with cold water or salt water
- Suck on ice chips or sugarless hard candies. Lemon drops & other sour candies are great for stimulating saliva production, as is sugarfree gum.

If these don't work, there are lots of over-the-counter artificial saliva products that come in sprays, lozenges, rinses, gel forms, including things like Aquoral, Biotene, Caphosol, NeutraSal, Entertainer's Secret, Mouth Kote, Numoisyn, Oasis, and SalivaSure. There are also store brands at many pharmacies. The choice among these is a matter of personal taste. They're all different formulations and may have different flavors, so try out a few to find the one that works best.

Notes: Also important, frequent dental care! Check-ups at least every 6 months to make sure that any problems are caught and addressed early.

TIP Sheet: Fall Prevention Checklist

The two major causes of falls are Health related changes such as certain medications, slow reflexes, poor eyesight and balance problems.

Dangerous situations in the home include slippery floors, poor lighting, electrical wires and cords in pathways, loose rugs, raised thresholds and clutter.

Most falls occur in bathrooms, bedrooms and on stairs. Use this checklist to help minimize the risk of falls in your home.

Approach to House

- ☐ Park close to your door.
- ☐ Be sure there is a clear pathway from the car to the door.
- ☐ Make sure the surface of the pathway is smooth and does not have cracks.
- ☐ Make sure there is a good light to show you the way to the door.
- ☐ If you have stairs, are the handrails steady and secure?
- ☐ Is the door wide enough to get through without straining?
- ☐ Make sure the door is easy to open and close.
- ☐ Paint the edges of outdoor steps and any steps that are especially narrow or uneven.

Living Room

- ☐ Is the seating adequate and safe?
- ☐ Can you get in and out of the furniture easily?
- ☐ Be sure the pathways are clear of electrical wires, clutter or cords.
- ☐ Be sure you have adequate lighting in the room.
- ☐ Be sure light switches are easy to reach and are at the entrance to the room.
- ☐ Make sure you have the telephone near and access to turn the television off and on without getting up from your chair.
- ☐ If you have floor rugs, remove them and wear non-slip, low heeled shoes or slippers with rubber grippers on the bottom. Do not walk around in stocking feet.

Kitchen

- ☐ Is the seating adequate and safe?
- ☐ Be sure there is good lighting both in the room and in the work areas.
- ☐ Move the items you use most often to shelves that you can easily reach without a step stool.
- ☐ Do not use a step stool. Ask someone to help you if you must reach for something.
- ☐ Are your appliances in good condition and accessible?

Bedroom

- ☐ Place a lamp close to the bed where it is easy to reach without getting out of bed.

- ◻ Be sure the path from the bed to the bathroom is clear of clutter and is lit by a nightlight.
- ◻ Put a nightlight in the bedroom and hallways.
- ◻ Remove any floor rugs.
- ◻ Have the telephone close to the bed so you can reach it without getting out of bed.
- ◻ Have the television remote near the bed to avoid getting up to turn it on and off.

Bathroom

- ◻ Clear the pathway to the bathroom of any clutter, wires or cords.
- ◻ Be sure to turn the lights on when you enter.
- ◻ Do you have grab bars in the shower, tub and toilet areas? If not, have these installed.
- ◻ Remove any bathroom area rugs.
- ◻ Use non-slip adhesive strips or bath mats with strong suction grip.
- ◻ Consider sitting on a chair or stool in the shower.
- ◻ Use an elevated toilet seat to make it easier to sit and stand.
- ◻ Is there a telephone in the bathroom close to floor or is safety device worn during showering?

Stairs and Steps

- ◻ Pick up things on stairs such as books, shoes, laundry or other objects.
- ◻ Be sure the stairway is well lit.
- ◻ Have glowing light switches installed at the top and bottom of the stairwell.
- ◻ Repair any broken or loose steps.
- ◻ Repair any broken or loose handrails.
- ◻ Paint a brightly colored strip on the top and bottom steps.

General Safety

- ◻ Do you have smoke detectors in place?
- ◻ Use helping devices such as walkers or canes if necessary.
- ◻ Store items you use often on the counter or in an easy to reach drawer.
- ◻ Wear skid-free shoes.
- ◻ Keep a flash light with a fresh battery in the bedroom, kitchen and living room in case of loss of power.
- ◻ Review medications with your doctor or pharmacist.
- ◻ Have your hearing and eyesight checked. Inner ear problems can affect balance and vision problems make it difficult to see potential fall hazards.
- ◻ Exercise regularly to improve balance, flexibility, strength and overall well-being.
- ◻ If you feel dizzy, sit down and allow enough time for the dizziness to clear. Stand up slowly and hold onto something steady.
- ◻ If a step stool is absolutely necessary, use one with high and steady handrails. Do not use wobbly stools or chairs to reach things.
- ◻ Think about wearing an alarm device that will bring help- in case you fall and can't get up.
- ◻ Be aware that alcohol affects your balance.

TIP Sheet: Medication Management

Taking your medication on time and correctly can help to improve your quality of life, and decrease disability. For most people, it's difficult to remember to take daily medications on time.

Tips for Remembering to Take Medication on Time:

- Pick a specific day of the week each week to put your pills in a 7-day medication organizer.
- Consider using a pill box with a timer.
- Use a cell phone timer to remember your medications.
- Take your medicine **before** you silence the alarm.
- Keep your pill box organizer is a place where you will see it daily (e.g., on the kitchen table, or on your dresser.
- Post a list of your medications with the strength, reason, and times you take each medication. **First**, take your pill, **then** check it off the list.
- Make it a point of noticing when you take your medication.
- Have your care-partner, aide, or family member help you to remember your medications.

Leaving Your Home:

• Have a pill container to bring with you when you leave the house.
• Bring extra medication in case you are away from home longer than expected.

Medication Safety:

• If your medication suddenly changes color, shape, or size, double-check to make sure you were given the correct medication. Call your pharmacy, or show your medicine to your pharmacist.
• Take unused, unneeded, or expired medication back to pharmacy.
• Find Prescription Drug Take-Back events in your community.
• Keep your medications separate from those of your other family members.
• Take your medication in a well-lighted area. Never take medications in the dark.

Notes: When traveling, make sure you have more than enough medication for your trip. ALWAYS keep your medications in your carry-on bag, never put them in your checked luggage. Bring your medication list with you, as well as contact information for your doctor and pharmacy in case of an emergency.

TIP Sheet: Orthostatic Hypotension

Orthostatic hypotension is when your blood pressure falls suddenly when standing up or changing position. This can put you at risk for fainting, falling, and injuries.

Symptoms of orthostatic hypotension include:

- Dizziness
- Lightheadedness
- Weakness
- Blurred vision
- Passing out, or fainting (syncope)

Tips for controlling orthostatic hypotension:

1. Change position slowly. For example, move slowly from laying down, to sitting on the edge of your bed, to standing.
2. Drink plenty of liquids. Drink six 8-ounce glasses of water daily.
3. Avoid caffeinated drinks. Caffeine will decrease blood volume and worsen orthostatic hypotension.
4. Elevate the head of your bed 30° at night.
5. Eat small, frequent meals.
6. Try isometric exercises before changing position or standing up.
   For example:
   Calf raises: while seated raise your heels, contract your calf muscle, and hold for 30 seconds. Repeat 3 times.
7. Consider using support stockings or an abdominal binder.
8. Talk to your healthcare provider about increasing salt in your diet.
9. Your healthcare provider may recommend medications like fludrocortisone (Florinef) or midrodrine (ProAmatine) to help control orthostatic hypotension. Take these medications as prescribed.

Notes: If you feel like you may faint

- **lie down** and **elevate your legs** above your heart

OR

- **sit down** and place your **head between your knees**.

TIP Sheet: Excess Saliva and Drooling

Sialorrhea is excess saliva in the mouth that can contribute to drooling.

People living with Parkinson's disease do not make more saliva. Instead, they swallow less frequently, causing pooling of saliva in the mouth.

Sialorrhea can be embarrassing, but it can also be a risk factor for aspiration pneumonia.

Tips for controlling sialorrhea:

1. Suck on sugar free hard candy. This will help remind you to swallow more frequently.

2. Chew sugar free gum.

3. Try consciously reminding yourself to swallow more frequently.

4. Ask your healthcare provider about speech therapy to strengthen the muscles around your lips.

5. Try exercises like blowing kisses, or drinking from a straw to strengthen the muscles around your lips.

6. Talk to your healthcare provider about possible medications including Atropine drops, and Glycopyrrolate to help reduce excess saliva.

7. Your healthcare provider may recommend botulinum toxin (Botox®) injections to help control sialorrhea. This is when a healthcare professional injects your parotid glands (salivary glands) with Botox® to reduce saliva production. This is not permanent, and results last for approximately 3-6 months.

Notes: The medications to help reduce excess saliva are not appropriate for everyone

TIP Sheet: Skin Changes in Parkinson's Disease

People living with Parkinson's disease may experience bothersome skin changes.

Seborrheic dermatitis:
Many people with Parkinson's develop seborrheic dermatitis. This is when tiny glands below the surface of the skin produce too much sebum (the natural oil of the skin). Seborrheic dermatitis may be bothersome, but it is not dangerous.

Symptoms of Seborrheic Dermatitis:

- oily, flaking, or reddened skin
- patches of white or yellowish oily scales commonly seen on the forehead, sides of the nose, scalp, and eyebrows
- dandruff on the scalp, in the ears, or in the eyebrows

Tips for dealing with Seborrheic Dermatitis:

1. Wash the skin twice daily with warm water and rinse with cold water.
2. Wash the scalp and face with an over-the-counter shampoo containing zinc or selenium.
3. If bothersome, talk with your healthcare provider about prescription lotions and shampoos.

Skin Cancer:

People living with Parkinson's disease have an increased risk of melanoma.

Tips for people with Parkinson's disease:

1. Have yearly skin checks with a dermatologist.
2. Seek shade, and wear hats, sun shirts, and wraparound sunglasses. Select sunscreen that has an SPF of 30 or higher, and provides UVA and UVB protection. Reapply sunscreen every two hours.
3. Report any new moles, abnormal moles, or other skin growths to your healthcare provider.
4. The **ABCDE** rule is a guide to the usual signs of melanoma. Talk to your doctor about any spots that have any of the following features:
   - **A**symmetry: Half of the mole does not match the other
   - **B**order: The edges are irregular, blurred, or ragged
   - **C**olor: The color is not the same all over
   - **D**iameter: The spot is larger than the size of a pencil eraser (6 mm)
   - **E**volving: The mole is changing in size, shape, or color

TIP Sheet: Getting a Good Night's Sleep

Steps for getting to sleep faster and staying asleep:

- Do something relaxing for at least 30 minutes before you go to bed. This may include reading, coloring, or listening to music.
- Engage in relaxation exercises or meditation before bed.
- Keep a regular sleep schedule. Go to bed at the same time each night and get up at the same time each morning.
- Exercise during the day, but avoid exercise after 8 PM.
- Try to get 7-8 hours of sleep per night.
- Avoid daytime naps. If you do nap, do it at the same time every day and limit it to 1 hour at the most. Do not nap after 3 PM.
- Avoid caffeine and other stimulants 6 hours before bedtime.
- Avoid alcohol because it can disrupt sleep.
- Sleep in a cool dark place. Do not read or watch television in bed.
- Avoid television, tablets, phones, and computers 1-2 hours before bed.
- If you cannot fall asleep after 20 minutes of lying in bed, get up, leave the bedroom, and something relaxing like reading, or listening to music.

Other tips:

- Try silky or satin sheets or pajamas to make moving in bed easier
- Avoid liquids for 3 hours before bedtime to prevent getting up to go to the bathroom at night
- Place a commode next to the bed to minimize effort if you do need to get up at night to use the bathroom.

Notes: Your healthcare provider may recommend a sleep study. This is a noninvasive overnight exam to monitor what is happening in your brain and body when you sleep. The test requires that you sleep at sleep center for the night.

TIP Sheet: Difficulty Swallowing

Dysphagia is when a person experiences difficulty or discomfort when swallowing.

It is important to tell your health care provider if you choke or cough when drinking liquids or eating food.

Swallowing trouble can put people at risk for potentially life-threatening aspiration pneumonia.

Tips for managing swallowing difficulties:

1. Sit upright at a 90° angle when eating. Remain upright for at least 30 minutes after eating.
2. Alternate between eating small bites of food and drinking small sips of liquid.
3. Consider using a thickener with thin fluids like water. You can buy Thick-It® over the counter at a drug store.
4. Avoid tough foods, like steak, that require excessive chewing.
5. Swallow medications with a spoonful of applesauce.
6. Ask your healthcare provider for a referral to see a speech language pathologist (SLP) for more tricks and tips to help with swallowing.
7. Take your time when eating and drinking.

Notes: Your health care provider may recommend a swallow study. This is a non-invasive, painless test where a speech language pathologist will watch you swallow while you eat food and drink liquids. After the test, the speech language pathologist may make recommendations to help you swallow more easily.

Tip Sheet: When to Call 911

When events happen at home or when new PD symptoms occur between visits, it can be hard deciding when to call your home study team, the Movement Disorders Clinic to page the on-call doctor, or 911. Hopefully this will be a handy tool.
If you are ever in doubt, call 911!

When to call the home visit study team (who will respond within 1-2 days):

- **You need refills or medication changes**:
  - If you feel your medication is not working or that you need a lower or higher dose.
- **You seem a little "off:"**
  - Your PD symptoms have gotten worse over the past week for no reason (i.e., missed doses, falls, etc.). Oftentimes, a urinary tract infection, cold, or constipation can be the cause.
  - You have sudden behavior changes: increased depression, hallucinations, new or increased obsessive-compulsive behaviors.
- You need to **reschedule or change** your **upcoming home visit.**
- **You were admitted to the hospital** and are updating us on your situation.
- We gave you a **referral** (in-home agency, therapist, etc.), and they are not getting back to you or are **not working out**.

When to call the Movement Disorders Clinic 312-563-2900 (who will respond within 1 day):

- **You fell, hit your head**, but did not pass out, bleed profusely, or show signs of concussion (nausea, vomiting, dizziness, or headache).
- You have noticed a **sudden increased number of falls** in the past week.
- You have called the study team **after business hours, on the weekend**, or no study team member has gotten back to you within 48 hours of leaving a message **and you would like to talk with an on-call physician**. If you call the clinic outside of business hours or on a weekend, your call will be forwarded to an answering service and the fellow on call will be paged.
- You need to **reschedule or change** your **upcoming clinic visit.**

When to call 911 (who will respond *immediately*):

- You have **new weakness, numbness, vision changes, trouble speaking or understanding, severe dizziness, or change in level of awareness**.
- You fell, hit your head, and either **passed out, started bleeding for more than one minute, or showed signs of a concussion** (see above).

- **You are feeling suicidal**, with or without a plan.
- **You are feeling unsafe at home** or someone is making you feel unsafe at home.
- If you feel like you have an emergency, please call 911 first!
